# Diagnostic performance of dual-energy CT for opportunistic detection of rotator cuff disease: a retrospective multireader study

**DOI:** 10.1186/s13244-025-02119-x

**Published:** 2025-10-27

**Authors:** Suwei Liu, Kai Ye, Yali Li, Aihui Di, Chenyu Jiang, Ming Ni, Huishu Yuan

**Affiliations:** Department of Radiology, Peking University Third Hospital, Beijing, China

**Keywords:** Rotator cuff, Dual energy, Computed tomography, Magnetic resonance imaging, Supraspinatus tendon

## Abstract

**Objectives:**

Multi-material decomposition (MMD), a key application of dual-energy computed tomography (DECT), has shown potential in musculoskeletal research. This study aimed to compare the diagnostic performance of DECT-based MMD with standard CT and MRI in detecting rotator cuff disease.

**Materials and methods:**

This retrospective study evaluated patients diagnosed with rotator cuff disease who underwent third-generation dual-source DECT and 3.0-T MRI within a 2-week interval between December 2023 and November 2024. Shoulder arthroscopy served as the reference standard. Six readers independently assessed rotator cuff tears and determined the degree of supraspinatus tendon diseases using standard CT, DECT-based MMD and MRI. Area under the curve (AUC), sensitivity, specificity, positive/negative predictive values and accuracy were calculated for the diagnosis of rotator cuff disease. Friedman test was used to analyze the radiologists’ diagnostic confidence across the three image types.

**Results:**

In total of 103 patients (mean age: 50.0 ± 15.6 years) underwent shoulder arthroscopy. MMD demonstrated a higher average AUC for diagnosing rotator cuff tears (88% vs. 65%, *p* < 0.001) and supraspinatus tendon disease (86% vs. 63%, *p* < 0.001) compared to standard CT. Its diagnostic performance for supraspinatus tendon disease (91% vs. 90%, *p* = 0.35) and full-thickness tears (95% vs. 93%, *p* = 0.11) was comparable to that of MRI.

**Conclusion:**

DECT-based MMD demonstrated superior diagnostic performance and reliability for detecting rotator cuff diseases compared to standard CT, with accuracy comparable to that of MRI in detecting supraspinatus tendon tears. DECT-based MMD offers a promising approach for the opportunistic detection of rotator cuff diseases.

**Critical relevance statement:**

Dual energy CT-based multi-material decomposition demonstrated accuracy comparable to that of MRI in detecting supraspinatus tendon tears, and may provide an alternative for patients with contraindications to MRI, facilitating early detection of injuries and accurate diagnosis of rotator cuff diseases.

**Key Points:**

- Dual energy (DE) CT multi-material decomposition (MMD) improves diagnostic performance for rotator cuff tears and supraspinatus tendon injuries.
- Radiologists with varying experience levels benefited from MMD, with experienced readers achieving MRI-level diagnostic performance.
- DECT MMD offers a promising alternative for patients with contraindications for MRI.

**Graphical Abstract:**

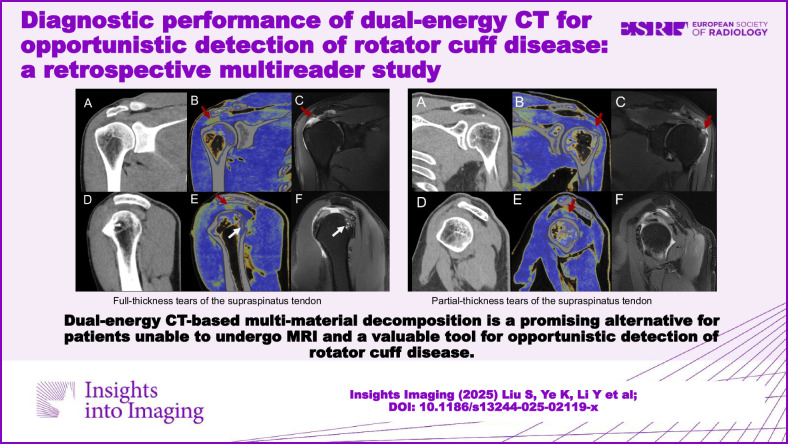

## Introduction

Shoulder pain is a common clinical symptom, often linked to various disorders, with rotator cuff disease being one of the most prevalent causes [1, 2]. Timely and accurate diagnosis is essential for guiding treatment decisions, improving outcomes, and minimizing healthcare costs [3–5].

Arthroscopy remains the gold standard for diagnosing rotator cuff disease because it provides direct visualization of intra-articular structures. However, MRI is the most accurate non-invasive imaging modality for assessing rotator cuff pathology [6, 7]. Although clinical examination detects certain full-thickness ruptures, MRI remains valuable for comprehensive evaluation, particularly in complex or equivocal cases. It is, however, limited by long waiting times, high costs, and motion artifacts, and it may be unsuitable for patients with certain implants or claustrophobia. Ultrasonography is a more accessible and cost-effective option, but operator dependence and reduced accuracy in patients with obesity or mobility limitations restrict its utility. CT arthrography is an alternative for evaluating the rotator cuff; however, it is often invasive and inconvenient [8].

Although standard CT is not routinely used as a first-line modality for the evaluation of rotator cuff disease because of its limited soft-tissue contrast, it remains widely utilized for assessing shoulder trauma, fractures, osteoarthritis, and degenerative joint conditions. Its rapid acquisition, broad availability, and ability to generate high-resolution 3D reconstructions make it valuable for evaluating bony structures and preoperative planning [9, 10]. Therefore, many patients undergo shoulder CT for non-rotator cuff-related indications, presenting an opportunity to explore rotator cuff pathology retrospectively through advanced CT techniques.

Dual-energy CT (DECT) is an advanced imaging technique that acquires data at two different X-ray energy levels, enabling material decomposition and enhanced tissue characterization based on the distinct attenuation properties of different substances [11–15]. Multi-material decomposition (MMD), a key application of DECT, has shown promising potential in musculoskeletal research, detecting conditions like bone marrow edema and intervertebral disc herniation, as well as evaluating injuries such as anterior cruciate ligament and Achilles tendon tears [16–20]. Therefore, DECT-based MMD may offer a promising approach for the opportunistic detection of rotator cuff disease.

This study aims to assess the diagnostic performance of DECT-based MMD in detecting rotator cuff disease, comparing it to standard CT and MRI and using arthroscopy as the reference standard.

## Materials and methods

This retrospective study was approved by the Institutional Review Board of Peking University Third Hospital (Approval Number: M2024188). Given the retrospective nature of the study and the use of de-identified patient data, the requirement for informed consent was waived by the IRB.

### Patient selection and study design

This retrospective study included patients who underwent shoulder DECT examinations between December 2023 and November 2024 for shoulder pain. Although these DECT scans were not originally performed for the evaluation of rotator cuff disease, all patients later underwent MRI and shoulder arthroscopy because of persistent symptoms or further clinical suspicion, which confirmed the presence of rotator cuff disease. These DECT scans were retrospectively reviewed to evaluate the diagnostic performance of DECT in detecting rotator cuff abnormalities. All patients included had noncontrast-enhanced DECT, MRI, and arthroscopic confirmation. The inclusion criteria were: (1) imaging examinations conducted within a 2-week period, and (2) complete arthroscopic findings. Exclusion criteria included: (1) history of previous shoulder surgery and (2) presence of shoulder joint tumors and tendon calcification. A total of 103 patients were included. The sample size was deemed sufficient based on comparable studies, which have utilized similar patient volumes to assess the diagnostic accuracy of imaging modalities for rotator cuff disease.

### DECT protocol

Noncontrast-enhanced CT was performed using a third-generation dual-source DECT system (Somatom NewForce; Siemens Healthineers). Two X-ray tubes operated at different kilovolt settings (Tube A: 100 kVp, 220 mAs; Tube B: Sn 150 kVp with a 0.64 mm tin filter, 138 mAs). The dual-energy protocol used a collimation width of 192 × 0.6 mm, a pitch of 0.6, and a rotation time of 500 ms, with tube current modulation enabled (CAREDose4D). The mean CT volume dose index was 20.5 ± 14.48 mGy (range: 16.5–27.8 mGy), and the mean dose-length product was 383.1 ± 63.7 mGy·cm (range: 263.9–513.6 mGy·cm). Patients were scanned in the supine position, headfirst, from the acromioclavicular joint to the inferior angle of the scapula, encompassing the scapula, proximal humerus, and the outer two-thirds of the clavicle.

### CT image reconstruction and post-processing

**Fig. 1:**
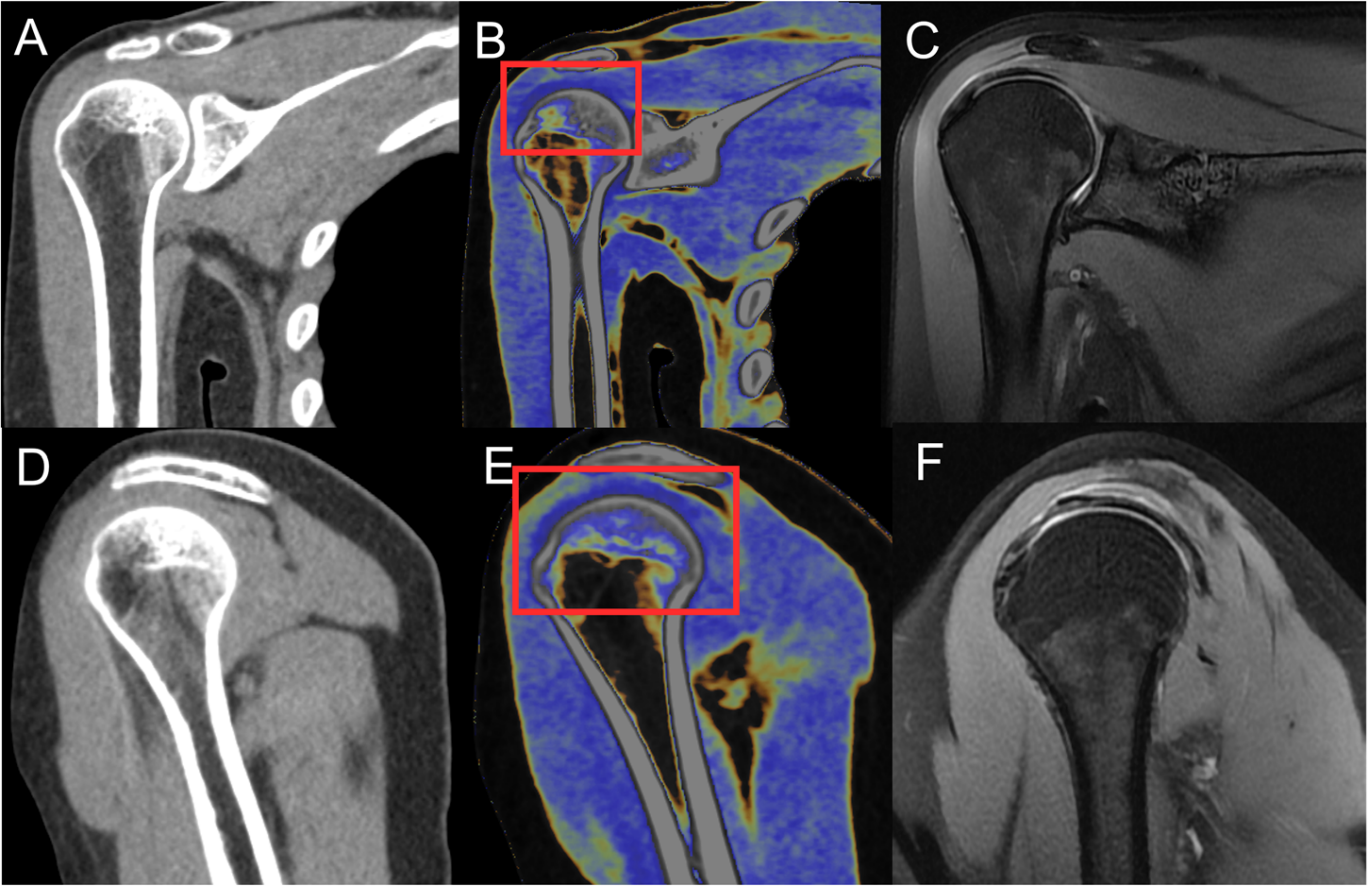
Images for a 26-year-old woman with a normal shoulder. **A**, **D** At standard CT, the rotator cuff is indistinct. **B**, **E** After MMD reconstruction images, the normal rotator cuff is dark blue (red rectangular). **C**, **F** Noncontrast MRI in oblique coronal and oblique sagittal positions demonstrates a normal rotator cuff on fat-suppressed proton density-weighted imaging. MMD, multi-material decomposition

The DECT scan data (100 kVp, Sn 150 kVp) were reconstructed using a medium-soft convolution kernel (Qr40) with advanced model-based iterative reconstruction (ADMIRE) at level 3. Mixed images (equivalent to standard CT) were generated on a SyngoVia^®^ VB30 workstation (Siemens, Erlangen). The commercially available dual-energy “Virtual Non-Contrast (VNC) application with a fat map” was utilized for MMD image generation; it was specifically used to generate tendon and ligament evaluation maps. These color-coded maps were generated based on material attenuation differences across dual-energy acquisitions. Normal tendons were visualized in dark blue colors, and tendon fiber discontinuity was shown in yellow (Fig. 1). Axial, oblique coronal, and oblique sagittal images were reconstructed using standard CT and MMD images. Oblique sagittal images were aligned parallel to the glenoid, and oblique coronal images were parallel to the supraspinatus muscle. Images were transmitted to the picture archiving and communication system (PACS) for further analysis.

### MRI protocol

MRI was performed using a 3.0-T scanner (uMR Omega, uMR 880i). Sequences included fat-suppressed proton density-weighted imaging (axial, oblique sagittal, and oblique coronal) with fast spin echo (FSE); reverse time (TR) = 3000–3200 ms, echo time (TE) = 65–80 ms, field of view (FOV) = 160 × 160 mm, matrix = 240 × 320, slice thickness = 3.0 mm, and slice gap = 0.3 mm. Oblique coronal T1-weighted imaging included using FSE with TR = 500–600 ms, TE = 16–20 ms, FOV = 160 × 160 mm, matrix = 256 × 256, slice thickness = 3.0 mm, and slice gap = 3.0 mm.

### Image evaluation

Image evaluation was performed using the picture archiving and communication system workstation (version 4.2; GE Healthcare). Arthroscopic findings, considered the gold standard, were used to record the presence or absence of rotator cuff tears (supraspinatus, infraspinatus, and subscapularis tendons) and to assess the degree of supraspinatus tendon injury, categorized as tendinopathy without tear, partial tear, or full-thickness tear.

Six radiologists with varying levels of experience in musculoskeletal imaging independently evaluated standard CT, MMD, and MRI series. The panel included H.Y. (chief physician specializing in musculoskeletal imaging, over 15 years of experience), K.Y. (board-certified radiologist, 8 years of experience), C.J. (radiology resident, 6 years of experience), M.N. (radiology resident, 6 years of experience), S.L. (radiology resident, 4 years of experience), and Y.L. (radiology resident, 3 years of experience). All readers were blinded to the arthroscopic results. Each reader independently reviewed standard CT, MMD, and MRI images, presented in random order, and assessed the presence of rotator cuff tears and the degree of supraspinatus tendon disease. An 8-week interval was maintained between evaluations to minimize recall bias. Diagnoses of rotator cuff diseases were recorded by the musculoskeletal radiologists; they included the following criteria: full-thickness tears (defined as complete fiber discontinuity with a high signal on FS-T2WI, or yellow area on MMD, or low-density areas on standard CT), partial tears (defined as partial fiber discontinuity with a high signal on FS-T2WI, or yellow area on MMD, or low-density areas on standard CT), and tendinopathy without tear (defined as fiber continuity with a localized high signal on FS-T2WI, or localized light yellow area on MMD, or localized low-density areas on standard CT).

Prior to image evaluation, all readers underwent training to ensure adherence to the assessment standards. Images from 10 individuals not involved in this study were used in the training, which included detailed instructions on CT image reconstruction methods, sample images, and scoring definitions. Readers adjusted window settings and scrolled through image stacks freely. Image quality, noise, and diagnostic confidence were rated on a five-point Likert scale (1 = unacceptable/cannot diagnose, 2 = less than average/poorly confident, 3 = average/diagnosis is probable, 4 = more than average/probably confident, 5 = excellent/absolutely confident) [21].

### Statistical analysis

Statistical analyses were performed using SPSS Statistics for Windows (version 26.0; IBM), MedCalc for Windows (version 13), and R software (version 4.4.0; https://www.r-project.org/). Statistical significance was set at *p* < 0.05.

To account for clustering of multiple tendons within individual patients, the method described by Genders et al [22] was applied to both individual and consensus readings when calculating diagnostic performance metrics. The 2*C chi-square test was used to compare the results of standard CT, MMD, and MRI images in the assessment of rotator cuff tears and the degree of supraspinatus tendon diseases. Sensitivity, specificity, positive and negative predictive values, accuracy, and area under the curve (AUC) were calculated for each reader for the diagnosis of rotator cuff disease. AUC values for standard CT, MMD, and MRI were compared using the Delong test, and the inter-reader agreement was assessed using weighted Fleiss’ kappa. Friedman test analyzed the radiologists’ diagnostic confidence across the three image types.

## Results

**Fig. 2:**
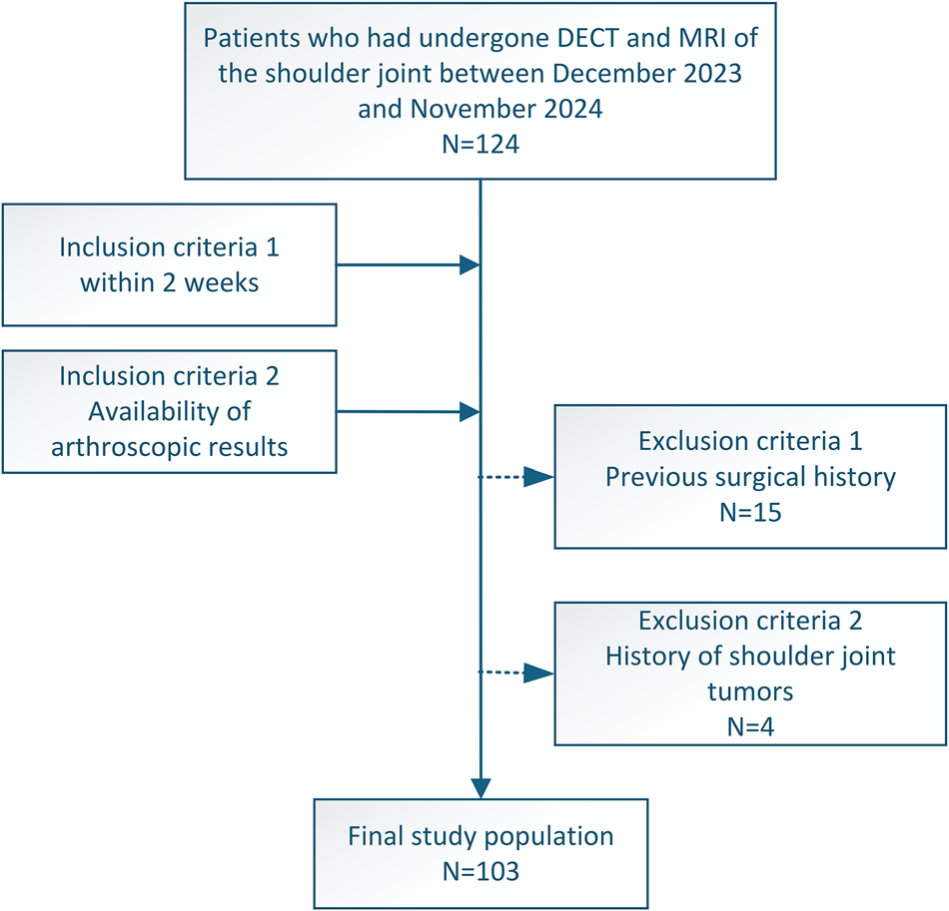
The flowchart shows the selection of the study population

A total of 103 patients (mean age: 50.0 ± 15.6 years; range: 15–75 years) were included in the study (Fig. 2), including 54 males (52.4%, 54/103; mean age: 46 ± 15.8 years; range: 17–75 years) and 49 females (47.6%, 49/103; mean age: 54 ± 14.4 years; age range: 15–73 years). Arthroscopy identified 65 supraspinatus, 15 infraspinatus, and 11 subscapularis tendon tears. The degree of supraspinatus tendon disease was classified as tendinopathy without tear in 38 patients (36.9%, 38/103), partial tears in 43 patients (41.7%, 43/103), and full-thickness tears in 22 patients (21.3%, 22/103). The average interval between all pairwise combinations of DECT, MRI, and arthroscopy was 5 days (range, 0–14 days) (Table 1).

**Table 1:** Patient characteristics

Characteristic	Value
Overall age (years)	50.0 ± 15.6 (15–75)*
Gender	
Female	49 (47.6) 54 ± 14.4 (15–73)*
Male	54 (52.4) 46 ± 15.8 (17–75)*
Side	
Right	68 (66.0)
Left	35 (34.0)
Arthroscopy result (*n*)	
Supraspinatus tendon tear	65 (63.1)
Infraspinatus tendon tear	15 (14.6)
Subscapularis tendon tear	11 (10.7)
Degree of supraspinatus tendon injury	
Tendinopathy	38 (36.9)
Partial tear	43 (41.7)
Full-thickness tear	22 (21.3)
Examination interval (day)	5 (0–14)

* Data are means ± standard deviation, with ranges in parentheses. Other than that, the data in parentheses are percentages. Among the tendinopathies, there were 8 cases of calcific tendinitis

### Standard CT, MMD, and MRI in rotator cuff tear diagnosis

**Fig. 3:**
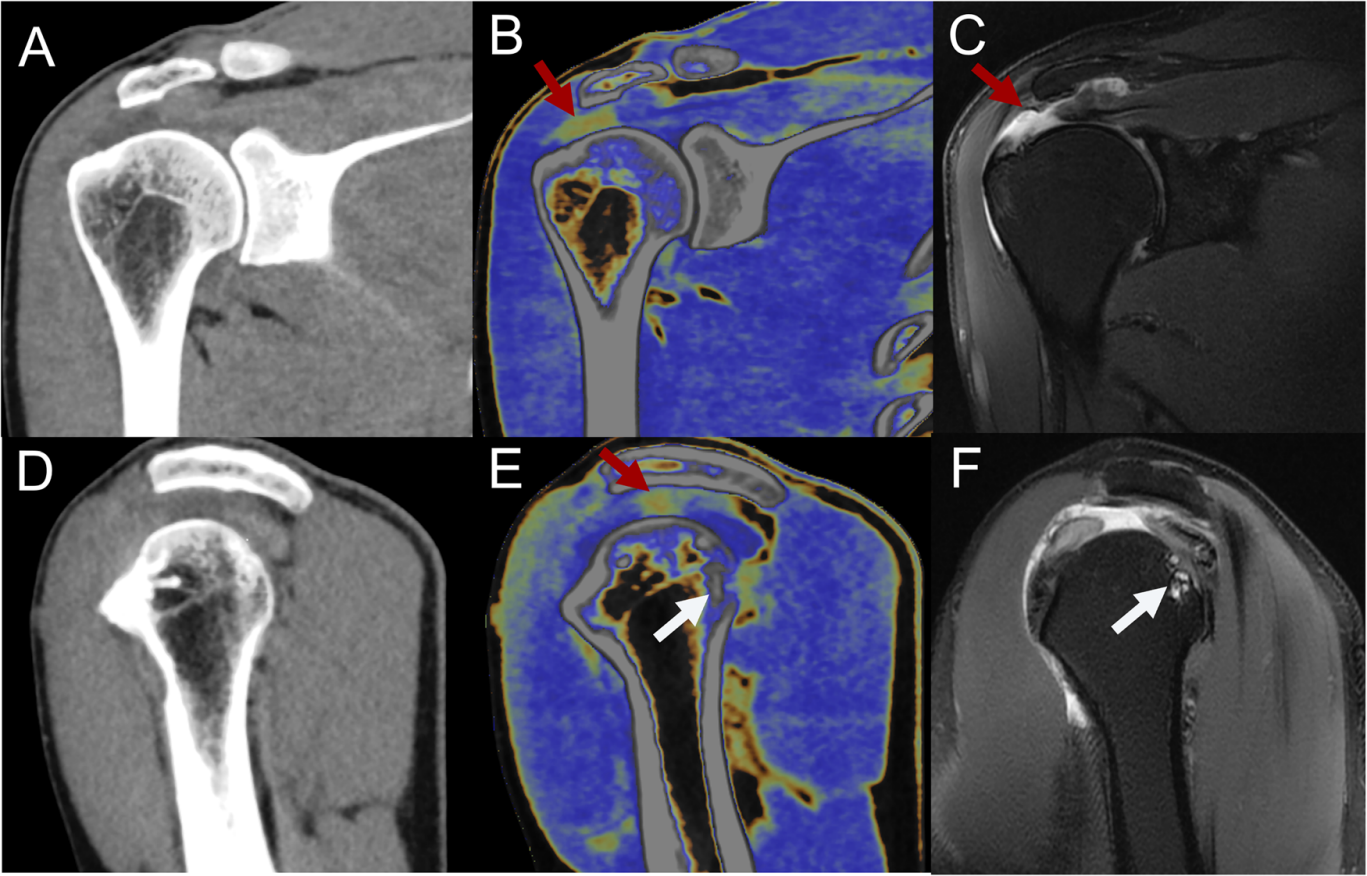
Images for a 56-year-old man presenting with acute severe right shoulder pain and swelling after exercise. **A**, **D** At standard CT, the supraspinatus tendon tear initially did not clearly show upon gray-scale reconstruction. **B**, **E** Supraspinatus tendon tears were detected by all readers after MMD reconstruction images were optimized for tendon analysis. **C**, **F** Noncontrast MRI in the oblique coronal and sagittal positions shows the supraspinatus tendon tear on fat-suppressed proton density-weighted imaging (red arrow). **E**, **F** Osteocortical discontinuity at the greater tuberosity of the humerus and subcortical cyst formation (white arrow). MMD, multi-material decomposition

**Fig. 4:**
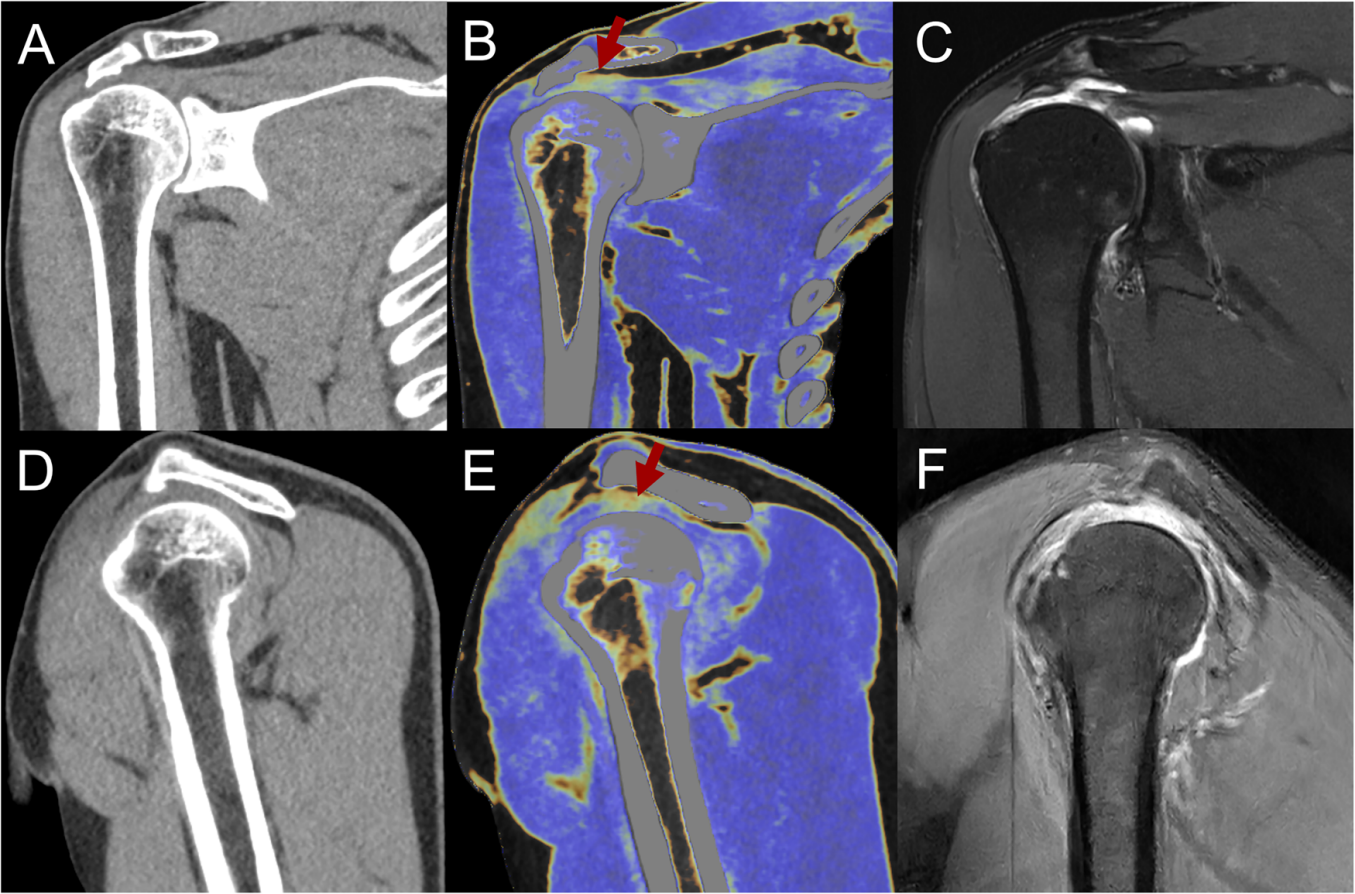
Images for a 73-year-old man experiencing chronic right shoulder pain with limited motion and a positive Neer impingement test. **A**, **D** At standard CT, acromion osteomalacia and subacromial gap narrowing. **B**, **E** After MMD reconstruction, images detected acromion impingement with supraspinatus, infraspinatus, and subscapularis tendon tears (red arrows). **C**, **F** Noncontrast MRI oblique coronal and sagittal views show fat-suppressed proton density-weighted images of superior acromial impingement with the rotator cuff full-thickness tear. MMD, multi-material decomposition

For supraspinatus tendon tears, MMD showed a significantly higher AUC compared to standard CT (90% vs. 66%, *p* < 0.001). There were no significant differences between the diagnostic performance of MRI and MMD (91% vs. 90%, *p* = 0.35) (Table 2, Fig. 3). The inter-reader agreements for standard CT, MMD, and MRI were 0.71, 0.76, and 0.78, respectively (*p* < 0.05). For infraspinatus tendon tears, MMD demonstrated a higher AUC compared to standard CT (80% vs. 60%, *p* < 0.001). Similarly, MMD exhibited superior performance for subscapularis tendon tears (81% vs. 62%, *p* < 0.001). Significant differences in diagnostic performance were observed between MRI and MMD for both infraspinatus and subscapularis tendon tears (90% vs. 80%; 89% vs. 81%, *p* < 0.05, respectively) (Table 2, Fig. 4). The inter-reader agreements for standard CT, MMD, and MRI were 0.57, 0.66, and 0.76, respectively, for infraspinatus tendon tears and 0.53, 0.59, and 0.80, respectively, for subscapularis tendon tears (*p* < 0.05).

**Table 2:** Diagnostic performance of rotator cuff tear and degree of supraspinatus tendon injury on standard CT, MMD reconstruction and MRI images

Parameter	Sensitivity (%)	Specificity (%)	Accuracy (%)	PPV (%)	NPV (%)	AUC(%)
Supraspinatus tendon tear^⁎†^						
SCT	132/390 (34) [29, 39]	226/228 (99) [97, 100]	358/618 (58) [54, 62]	132/134 (99) [95, 100]	226/484 (47) [42, 51]	66 [63, 70]
MMD	325/390 (83) [79, 88]	219/228 (96) [93, 98]	544/618 (88) [85, 90]	325/334 (97) [95, 99]	219/284 (77) [72, 82]	90 [87, 92]
MRI	355/390 (91) [88, 93]	207/228 (91) [86, 94]	562/618 (91) [88, 93]	355/376 (94) [92, 96]	207/242 (86) [81, 89]	91 [89, 93]
Infraspinatus tendon tear^⁎†※^						
SCT	18/90 (20) [13, 29]	526/527 (99) [98, 100]	544/618 (88) [85, 90]	18/19 (95) [75, 99]	526/598 (88) [85, 90]	60 [56, 64]
MMD	54/90 (60) [50, 70]	526/527 (99) [98, 100]	580/618 (94) [92, 96]	54/55 (98) [90, 100]	526/562 (94) [91, 95]	80 [75, 85]
MRI	73/90 (81) [72, 88]	525/527 (99) [99, 100]	598/618 (97) [95, 98]	73/75 (97) [91, 99]	525/542 (97) [95, 98]	90 [86, 94]
Subscapularis tendon tear^⁎†※^						
SCT	16/66 (24) [16, 36]	549/552 (99) [98, 100]	565/618 (91) [89, 93]	16/19 (84) [62, 95]	549/599 (92) [89, 94]	62 [57, 68]
MMD	42/66 (64) [52, 74]	547/552 (99) [98, 100]	589/618 (95) [93, 97]	42/47 (89) [77, 95]	547/571 (96) [94, 97]	81 [76, 87]
MRI	52/66 (79) [68, 87]	549/552 (99) [98, 100]	601/618 (97) [96, 98]	52/55 (95) [85, 98]	549/563 (98) [96, 99]	89 [84, 94]
Supraspinatus tendon tendinopathy^⁎†※^						
SCT	222/228 (97) [94, 99]	190/390 (49) [44, 54]	412/618 (67) [63, 70]	222/422 (53) [47, 57]	190/196 (97) [93, 99]	73 [70, 76]
MMD	216/228 (95) [91, 97]	339/390 (87) [83, 90]	555/618 (90) [87, 92]	216/267 (81) [76, 85]	339/351 (97) [94, 98]	91 [87, 94]
MRI	212/228 (93) [89, 96]	370/390 (95) [92, 97]	582/618 (94) [92, 96]	212/232 (91) [87, 94]	370/386 (96) [93, 97]	94 [91, 97]
Supraspinatus tendon partial tear^⁎†※^						
SCT	17/258 (7) [4, 9]	330/360 (92) [88, 94]	347/618 (56) [52, 60]	17/47 (36) [24, 51]	330/571 (58) [52, 63]	49 [43, 55]
MMD	163/258 (63) [57, 68]	338/360 (94) [91, 96]	501/618 (81) [78, 84]	163/185 (88) [83, 92]	338/433 (78) [74, 82]	79 [74, 83]
MRI	225/258 (87) [83, 91]	334/360 (93) [90, 95]	559/618 (91) [88, 92]	225/251 (90) [85, 93]	334/367 (91) [88, 94]	90 [87, 94]
Supraspinatus tendon full-thickness tear^⁎†^						
SCT	69/132 (52) [43, 61]	474/486 (97) [95, 99]	543/618 (88) [85, 90]	69/81 (85) [75, 92]	474/537 (88) [85, 91]	75 [70, 79]
MMD	116/132 (88) [81, 93]	476/486 (98) [96, 99]	592/618 (96) [94, 97]	116/126 (92) [86, 96]	476/492 (97) [95, 98]	93 [90, 96]
MRI	122/132 (92) [87, 96]	476/486 (98) [96, 99]	598/618 (97) [95, 98]	122/132 (92) [86, 96]	476/486 (98) [96, 99]	95 [93, 98]

Arthroscopic results as the gold standard. Denominators represent the total number with respect to the respective statistical measure. Data in brackets are 95% confidence intervals

*NPV* negative predictive value, *PPV* positive predictive value, *AUC* area under the curve, *MMD* multi-material decomposition

^⁎^ Statistically different values from CT vs. MMD

^†^ Statistically different values from CT vs. MRI

^※^ Statistically different values from MMD vs. MRI

### Standard CT, MMD, and MRI in the degree of supraspinatus tendon disease

**Fig. 5:**
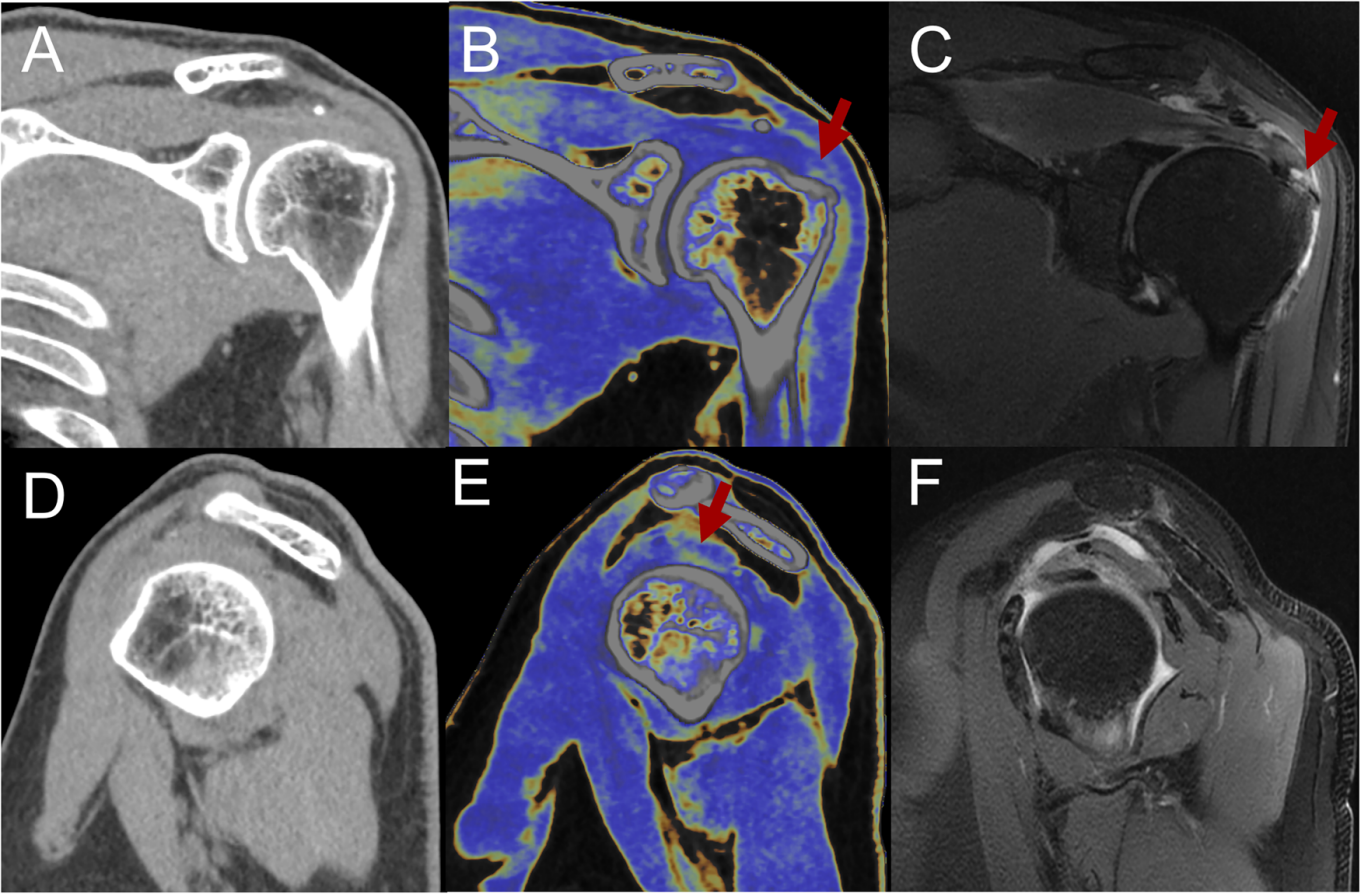
Images for a 60-year-old man with left shoulder pain and limited motion for several months. **A**, **D** At standard CT, the supraspinatus tendon partial tear was initially not visible on gray-scale reconstruction. **B**, **E** Supraspinatus tendon partial tears were detected after MMD reconstruction images were optimized for tendon analysis. **C**, **F** Noncontrast MRI in oblique coronal and oblique sagittal views shows supraspinatus tendon partial tears on fat-suppressed proton density-weighted images (red arrow). MMD, multi-material decomposition

For full-thickness tears of the supraspinatus tendon, MMD achieved a significantly higher AUC compared to standard CT (93% vs. 75%, *p* < 0.001). No significant differences were observed between the diagnostic performance of MMD and MRI (93% vs. 95%, *p* = 0.11) (Table 2, Fig. 3). The inter-reader agreements for standard CT, MMD, and MRI were 0.71, 0.89, and 0.95, respectively (*p* < 0.05). For partial tears and tendinopathy without a tear, MMD demonstrated higher AUCs compared to standard CT (91% vs. 73%; 79% vs. 49%, *p* < 0.001, respectively). Significant differences in diagnostic performance were noted between MRI and MMD for both partial tears and tendinopathy without tear (94% vs. 91%; 90% vs. 79%, *p* < 0.05, respectively) (Table 2, Fig. 5). The inter-reader agreements for standard CT, MMD, and MRI were 0.74, 0.79, and 0.86, respectively, for tendinopathy without tear, and 0.71, 0.76, and 0.83, respectively, for partial tears (*p* < 0.05).

MMD significantly improved the overall AUC for each reader in diagnosing rotator cuff tears and the degree of supraspinatus tendon disease compared to standard CT (88% vs. 65%; 86% vs. 63%, *p* < 0.001, respectively). However, MMD did not achieve the diagnostic performance of MRI (93% vs. 88%; 93% vs. 86%, *p* < 0.05, respectively). Notably, the most experienced reader achieved a diagnostic performance comparable to that of an MRI for detecting rotator cuff tears, with AUCs of 96% vs. 94% (*p* = 0.36) and for the degree of supraspinatus tendon disease at 94% vs. 92% (*p* = 0.34) (Tables 3 and 4).

**Table 3:** Comparison of the diagnostic performance of all readers for the detection of rotator cuff tear

Parameter	Sensitivity (%)	Specificity (%)	Accuracy (%)	PPV (%)	NPV (%)	AUC
Average^⁎※^						
SCT	166/546 (30) [21, 39]	1302/1308 (99) [97, 99]	1468/1854 (79) [74, 84]	166/172 (97) [82, 99]	1302/1682 (77) [72, 82]	65 [60, 70]
MMD	421/546 (77) [68, 85]	1293/1308 (99) [97, 99]	1714/1854 (92) [89, 95]	421/436 (97) [90, 99]	1293/1418 (91) [87, 95]	88 [84, 92]
MRI	480/546 (88) [80, 94]	1282/1308 (98) [96, 99]	1762/1854 (95) [92, 97]	480/506 (95) [88, 98]	1282/1348 (95) [91, 98]	93 [90, 97]
Reader 1^⁎†※^						
SCT	20/91 (22) [15, 32]	216/218 (99) [97, 99]	236/309 (76) [71, 81]	20/22 (91) [72, 97]	216/287 (75) [70, 80]	61 [57, 65]
MMD	67/91 (74) [64, 81]	213/218 (97) [95, 99]	280/309 (90) [87, 93]	67/72 (93) [85, 97]	213/237 (90) [85, 93]	85 [81, 90]
MRI	74/91 (85) [72, 88]	211/218 (97) [94, 98]	285/309 (92) [89, 95]	74/81 (91) [83, 96]	211/228 (92) [88, 95]	89 [85, 93]
Reader 2^⁎†※^						
SCT	26/91 (29) [20, 29]	216/218 (99) [97, 99]	242/309 (78) [73, 83]	26/28 (93) [76, 99]	216/281 (77) [71, 82]	64 [59, 69]
MMD	64/91 (70) [60, 79]	215/218 (99) [96, 99]	279/309 (90) [86, 93]	64/67 (96) [87, 99]	215/242 (89) [84, 93]	84 [80, 89]
MRI	76/91 (84) [74, 90]	213/218 (98) [95, 99]	289/309 (94) [90, 96]	76/81 (94) [86, 98]	213/228 (93) [89, 96]	91 [87, 95]
Reader 3^⁎†※^						
SCT	27/91 (30) [21, 40]	217/218 (99) [97, 99]	244/309 (79) [74, 83]	27/28 (96) [82, 99]	217/281 (77) [72, 82]	65 [60, 70]
MMD	65/91 (71) [61, 80]	216/218 (99) [97, 99]	281/309 (91) [87, 94]	65/67 (97) [90, 99]	216/242 (89) [85, 93]	85 [81, 90]
MRI	81/91 (89) [81, 95]	214/218 (98) [95, 99]	295/309 (95) [93, 98]	81/85 (95) [88, 99]	214/224 (96) [92, 98]	94 [90, 97]
Reader 4^⁎†※^						
SCT	29/91 (32) [22, 42]	217/218 (99) [97, 99]	246/309 (80) [75, 84]	29/30 (96) [83, 99]	217/279 (78) [72, 83]	66 [61, 71]
MMD	70/91 (77) [67, 85]	215/218 (99) [96, 99]	285/309 (92) [89, 95]	70/73 (96) [88, 99]	215/236 (91) [87, 94]	88 [83, 92]
MRI	80/91 (88) [79, 94]	216/218 (99) [97, 99]	296/309 (96) [93, 98]	80/82 (97) [91, 99]	216/227 (95) [91, 98]	93 [90, 97]
Reader 5^⁎†※^						
SCT	29/91 (32) [22, 42]	218/218 (100) [98, 100]	247/309 (80) [75, 84]	29/29 (100) [88, 100]	218/280 (78) [73, 83]	66 [61, 71]
MMD	74/91 (81) [72, 89]	216/218 (99) [97, 99]	290/309 (94) [91, 96]	74/76 (97) [91, 99]	216/233 (93) [89, 96]	90 [86, 94]
MRI	84/91 (92) [85, 97]	214/218 (98) [95, 99]	298/309 (96) [94, 98]	84/88 (95) [89, 99]	214/221 (97) [94, 99]	95 [92, 98]
Reader 6^⁎†^						
SCT	35/91 (38) [28, 49]	218/218 (100) [98, 100]	253/309 (82) [77, 86]	35/35 (100) [90, 100]	218/274 (80) [74, 84]	69 [64, 74]
MMD	81/91 (89) [81, 95]	218/218 (100) [98, 100]	299/309 (96) [94, 98]	81/81 (100) [96, 100]	218/228 (96) [92, 98]	94 [91, 98]
MRI	85/91 (93) [86, 98]	214/218 (98) [95, 99]	299/309 (96) [94, 98]	85/89 (96) [89, 99]	214/220 (97) [94, 99]	96 [93, 99]

Diagnostic accuracy of standard CT and MMD reconstructions for the detection of rotator cuff tear with Arthroscopic results as the gold standard, taking into account clustering. Notably, the most experienced reader (reader 6, chief physician specializing in musculoskeletal imaging, over 10 years) was able to achieve similar diagnostic performance in the detection of rotator cuff tear by using MMD as with MRI. Reader 1 had 1 year of experience, reader 2 had 3 years, reader 3 had 3 years, reader 4 had 5 years, and reader 5 had 6 years. Denominators represent the total number with respect to the respective statistical measure

*NPV* negative predictive value, *PPV* positive predictive value, *AUC* area under the curve, *MMD* multi-material decomposition

^⁎^ Statistically different values from CT vs. MMD

^†^ Statistically different values from CT vs. MRI

^※^ Statistically different values from MMD vs. MRI

**Table 4:** Comparison of the diagnostic performance of all readers for the detection of the degree of supraspinatus tendon injury

Parameter	Sensitivity (%)	Specificity (%)	Accuracy (%)	PPV (%)	NPV (%)	AUC (%)
Average^⁎†※^						
SCT	310/618 (50) [40, 60]	937/1236 (76) [69, 82]	1247/1854 (67) [62, 72]	310/609 (51) [41, 61]	937/1245 (75) [69, 81]	61 [55, 66]
MMD	495/618 (80) [71, 87]	1153/1236 (93) [89, 96]	1648/1854 (89) [85, 92]	495/578 (86) [77, 92]	1153/1276 (90) [86, 94]	84 [80, 89]
MRI	559/618 (90) [84, 95]	1180/1236 (95) [92, 98]	1739/1854 (94) [91, 96]	559/615 (91) [84, 96]	1180/1239 (95) [91, 98]	91 [87, 96]
Reader 1^⁎†※^						
SCT	49/103 (48) [38, 58]	152/206 (74) [67, 80]	201/309 (65) [59, 70]	49/103 (48) [38, 58]	152/206 (74) [67, 80]	61 [55, 66]
MMD	78/103 (75) [66, 84]	191/206 (93) [88, 96]	269/309 (87) [82, 91]	78/93 (84) [75, 91]	191/216 (88) [83, 92]	84 [80, 89]
MRI	90/103 (87) [79, 93]	193/206 (94) [89, 97]	283/309 (92) [88, 94]	90/103 (87) [79, 93]	193/206 (94) [89, 97]	91 [87, 94]
Reader 2^⁎†※^						
SCT	54/103 (52) [42, 64]	157/206 (76) [70, 82]	211/309 (68) [63, 73]	54/103 (52) [42, 62]	157/206 (76) [70, 82]	64 [59, 70]
MMD	78/103 (76) [66, 84]	186/206 (90) [85, 94]	264/309 (85) [81, 89]	78/98 (80) [70, 87]	186/211 (88) [83, 92]	83 [78, 88]
MRI	92/103 (89) [82, 95]	194/206 (94) [90, 97]	286/309 (93) [89, 95]	92/104 (88) [81, 94]	194/205 (95) [91, 97]	92 [88, 95]
Reader 3^⁎†※^						
SCT	48/103 (47) [37, 57]	153/206 (74) [68, 80]	201/309 (65) [59, 70]	48/101 (47) [37, 58]	153/208 (74) [67, 79]	60 [55, 66]
MMD	77/103 (75) [65, 83]	188/206 (91) [87, 95]	265/309 (86) [81, 89]	77/95 (81) [72, 88]	188/214 (89) [83, 92]	83 [78, 88]
MRI	93/103 (90) [83, 95]	196/206 (95) [91, 98]	289/309 (94) [90, 96]	93/103 (90) [83, 95]	196/206 (95) [91, 98]	93 [89, 96]
Reader 4^⁎†※^						
SCT	52/103 (50) [40, 60]	155/206 (75) [69, 81]	207/309 (67) [61, 72]	52/103 (50) [40, 60]	155/206 (75) [69, 81]	63 [57, 69]
MMD	84/103 (81) [73, 89]	191/206 (93) [88, 96]	275/309 (89) [85, 92]	84/99 (85) [76, 91]	191/210 (91) [86, 94]	87 [83, 91]
MRI	95/103 (92) [85, 96]	198/206 (96) [92, 98]	293/309 (95) [92, 97]	95/103 (92) [85, 97]	198/206 (96) [92, 98]	94 [91, 97]
Reader 5^⁎†※^						
SCT	51/103 (50) [40, 60]	156/206 (76) [69, 81]	207/309 (67) [61, 72]	51/101 (50) [40, 61]	156/208 (75) [69, 81]	63 [57, 68]
MMD	88/103 (85) [77, 92]	196/206 (95) [91, 98]	284/309 (92) [88, 95]	88/98 (90) [82, 95]	196/211 (93) [89, 96]	90 [87, 94]
MRI	95/103 (92) [85, 97]	199/206 (97) [93, 99]	294/309 (95) [92, 97]	95/102 (93) [86, 97]	199/207 (96) [93, 98]	94 [92, 97]
Reader 6^⁎†^						
SCT	56/103 (54) [44, 64]	164/206 (80) [73, 85]	220/309 (71) [66, 76]	56/98 (57) [47, 67]	164/211 (78) [71, 83]	67 [61, 73]
MMD	90/103 (87) [79, 93]	201/206 (97) [94, 99]	291/309 (94) [91, 97]	90/95 (95) [88, 98]	201/214 (94) [90, 97]	92 [89, 96]
MRI	94/103 (91) [84, 96]	200/206 (97) [94, 99]	294/309 (95) [92, 97]	94/100 (94) [87, 98]	200/209 (96) [92, 98]	94 [91, 97]

Diagnostic accuracy of standard CT and MMD reconstructions for the detection of rotator cuff tear with Arthroscopic results as the gold standard, taking into account clustering. Notably, the most experienced reader (reader 6, chief physician specializing in musculoskeletal imaging, over 10 years) was able to achieve similar diagnostic performance in the detection of rotator cuff tear by using MMD as with MRI. Reader 1 had 1 year of experience, reader 2 had 3 years, reader 3 had 3 years, reader 4 had 5 years, and reader 5 had 6 years. Denominators represent the total number with respect to the respective statistical measure

*NPV* negative predictive value, *PPV* positive predictive value, *AUC* area under the curve, *MMD* multi-material decomposition

^⁎^ Statistically different values from CT vs. MMD

^†^ Statistically different values from CT vs. MRI

^※^ Statistically different values from MMD vs. MRI

### Diagnostic confidence

**Fig. 6:**
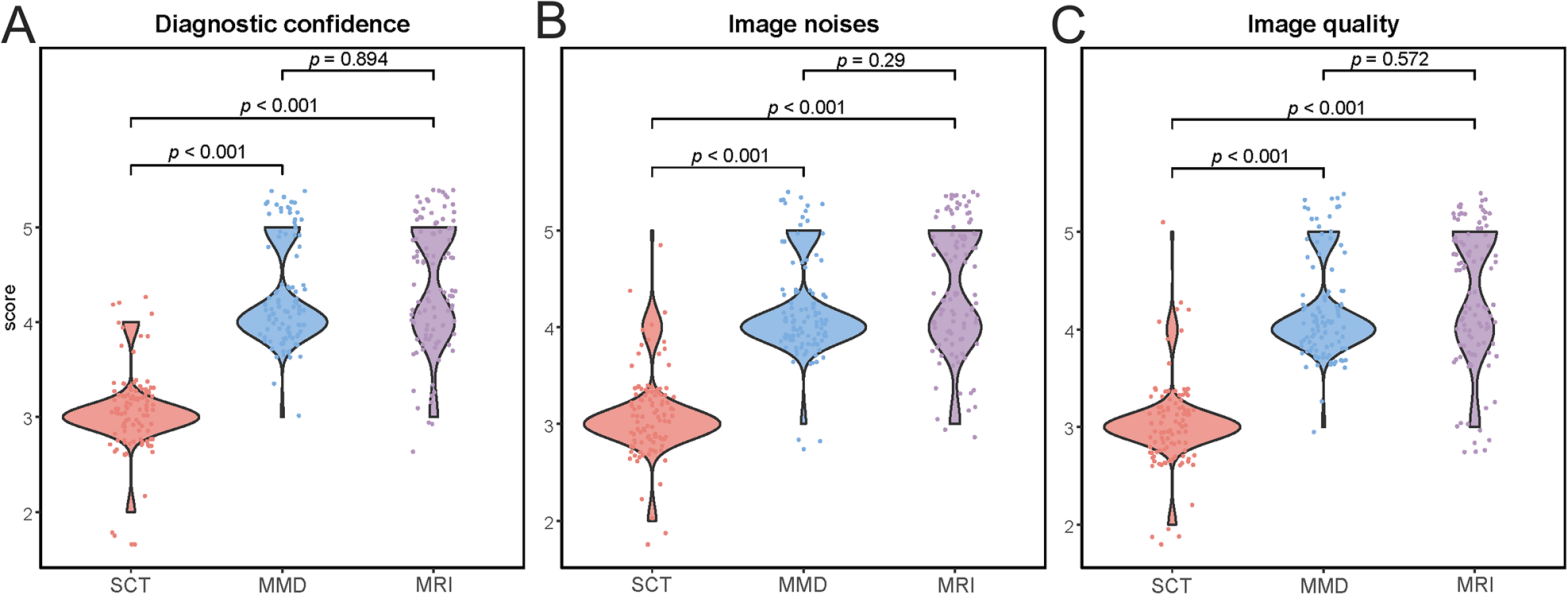
Violin and scatter plots demonstrate qualitative assessment of (**A**) diagnostic confidence, (**B**) amount of noise, and (**C**) image quality of MRI, standard CT, and MMD reconstructions in the study. Scatter plots show the distribution of scores. MMD reconstruction images achieved significantly higher mean scores than standard CT images for all categories (all *p* < 0.001). Regarding comparison of optimized MMD reconstructions and MRI series, no significant difference was found in diagnostic confidence (*p* = 0.894), amount of noise (*p* = 0.29), and image quality (*p* = 0.572). SCT, standard CT; MMD, multi-material decomposition

Regarding diagnostic confidence, all readers exhibited slightly higher confidence with MRI (mean score: 4.38) compared to MMD (mean score: 4.25; *p* = 0.89) (Fig. 6), while diagnostic confidence for standard CT was significantly lower (mean score: 3.05) (*p* < 0.001). The inter-reader agreement was good for MRI (*k* = 0.79), MMD (*k* = 0.70), and standard CT (*k* = 0.56).

MRI had a higher mean score of 4.37 for image noise compared to MMD (4.17; *p* = 0.29). Compared with standard CT (mean score: 3.09), both MRI and MMD showed significant differences (*p* < 0.001). The inter-reader agreement was excellent for MRI (*k* = 0.89) and MMD (*k* = 0.88), and good for standard CT (*k* = 0.75).

Regarding image quality, MRI received a mean score of 4.37, and MMD scored 4.20; however, the difference in ratings was not significant (*p* = 0.57). In contrast, standard CT had the lowest mean score at 3.04 (*p* < 0.001). Inter-reader agreement was excellent for MRI (*k* = 0.88) and MMD (*k* = 0.86), but good for standard CT (*k* = 0.75).

## Discussion

Standard CT has limited diagnostic capability for rotator cuff injuries [10, 23]. Our study found that DECT-based MMD significantly improved diagnostic performance for rotator cuff disease compared with standard CT, achieving an improvement exceeding 20%, suggesting its potential as a promising approach for detecting rotator cuff disease.

Our findings align with previous studies showing the capability of DECT in enhancing soft-tissue characterization through dual-energy material decomposition [24–26]. Similar improvements in tendon assessment have been reported in evaluating Achilles tendon injuries using MMD [20]. While the Achilles tendon has a more homogeneous structure, the current study extends MMD application to the more anatomically complex rotator cuff, reinforcing its potential utility across different tendon groups. Moreover, DECT has been used to quantitatively assess rotator cuff fatty infiltration, with fat fraction measurements strongly correlating with the Goutallier classification (ρ = 0.92, *p* < 0.0001) [27]. DECT virtual monochromatic imaging has demonstrated a diagnostic accuracy for supraspinatus tendon tears (85%) comparable to that of MRI (90%) and significantly higher than standard CTs (76%), further supporting its clinical relevance in tendon pathology [28]. Thus, our results align with prior research demonstrating that DECT improves the visualization of edema and soft-tissue lesions by exploiting differences in attenuation at different energy levels [29–31]. However, unlike MRI, DECT is associated with ionizing radiation exposure; nevertheless, the dose applied in this study remained within clinically acceptable limits and was comparable to that of conventional CT.

In this study, on comparing with MRI, DECT-based MMD achieved comparable diagnostic accuracy for supraspinatus tendon and full-thickness tears; however, it remained inferior for infraspinatus and subscapularis tendon injuries. This aligns with previous MRI studies reporting higher sensitivity for partial-thickness and subtle degenerative changes [32–34]. The inferior performance of MMD in these subgroups may be attributable to the small sample size, low tendon density, and overlapping anatomical structures. Nevertheless, DECT-based MMD showed substantial benefits for readers of varying experience levels, with the most experienced readers achieving diagnostic accuracy approaching that of MRI. This observation parallels prior work in musculoskeletal radiology, emphasizing the influence of reader expertise on advanced imaging interpretation [35].

The novelty of our study lies in being the first to systematically evaluate DECT-based MMD for rotator cuff diseases and directly compare its performance with standard CT and MRI in the same cohort. Clinically, our results suggest that MMD could also serve as a valuable opportunistic diagnostic tool in patients undergoing CT for other shoulder-related indications, potentially enabling early diagnosis and improved management. The technique’s capacity to enhance tissue differentiation may be especially beneficial in settings with limited MRI availability and in patients for whom MRI is contraindicated, broadening its potential clinical applicability.

Our study had several limitations. First, as a retrospective single-center study, the generalizability and external validity of our findings are limited. In particular, the relatively small number of cases involving infraspinatus and subscapularis tendon injuries may have led to an insufficient sample size for accurately evaluating diagnostic performance in these subgroups. Second, although MMD demonstrated high accuracy in detecting full-thickness rotator cuff tears, its ability to identify partial-thickness tears and subtle tendon degeneration—conditions more frequently encountered in clinical practice—remains limited. Therefore, MRI remains essential for comprehensive evaluation in such cases. Third, the CT scans used in this study were not originally acquired for rotator cuff assessment. Nevertheless, our results support the potential role of DECT as an opportunistic diagnostic tool, particularly in patients undergoing CT for other shoulder-related indications or those unable to undergo MRI. Finally, our findings are specific to the CT system and post-processing software used, which may limit generalizability across different platforms.

In conclusion, DECT-based MMD significantly improves diagnostic performance for rotator cuff tears compared to standard CT, approaching MRI’s performance for supraspinatus tendon and full-thickness tears. Furthermore, radiologists with varying levels of experience benefited from MMD. While radiation exposure remains a consideration, particularly in comparison with MRI, DECT-MMD offers substantial clinical value as an alternative or adjunct in specific patient populations. Additionally, it can opportunistically detect rotator cuff tears in patients undergoing CT for other indications. Future research should focus on dose-reduction strategies, multicenter validation, and integration with emerging technologies, such as photon-counting CT, to further enhance its diagnostic potential in shoulder disease evaluation.

## Data Availability

The datasets generated or analyzed during the study are available from the corresponding author on reasonable request.

## Abbreviations

AUC: Area under the curve
DECT: Dual-energy computed tomography
FOV: Field of view
MMD: Multi-material decomposition
MRI: Magnetic resonance imaging
NPV: Negative predictive value
PPV: Positive predictive value
